# Distinct clinical pattern of colorectal cancer patients with POLE mutations: A retrospective study on real-world data

**DOI:** 10.3389/fgene.2022.963964

**Published:** 2022-11-21

**Authors:** Miao Jiang, Yongliang Jia, Jinming Han, Jianxiang Shi, Chang Su, Rui Zhang, Menglu Xing, Shuiling Jin, Hong Zong

**Affiliations:** ^1^ Department of Oncology, The First Affiliated Hospital of Zhengzhou University, Zhengzhou, Henan, China; ^2^ BGI College and Henan Institute of Medical and Pharmaceutical Sciences, Zhengzhou University, Zhengzhou, Henan, China; ^3^ Precision Medicine Center, Henan Institute of Medical and Pharmaceutical Sciences, Zhengzhou University, Zhengzhou, China

**Keywords:** colorectal cancer, POLE mutation, polymerase epsilon, immunotherapy, prognosis

## Abstract

**Objective:** Studies have demonstrated an association between somatic POLE exonuclease domain mutations (EDMs) and the prognosis of colorectal cancer (CRC). However, the prognostic value of POLE non-EDMs remains unclear. This retrospective study aimed to explore the possible relationships between POLE mutation subtypes and CRC prognosis.

**Methods:** The 272 CRC patients from the First Affiliated Hospital of Zhengzhou University (ZZ cohort) and 499 CRC patients from The Cancer Genome Atlas database (TCGA cohort) were retrospectively collected. The cases were divided into subgroups based on POLE mutation sites and microsatellite instability (MSI) status. The continuous variables were compared among three subgroups with Kruskal-Wallis tests. Pairwise comparisons between three groups were performed by Bonferroni correction method, and adjusted *p* < 0.05 was considered statistically significant. The categorical variables were compared with Chi-square test and Fisher’s exact test. The Kaplan—Meier curves and Cox regression models were conducted to evaluate prognostic values of POLE mutations.

**Results:** In the ZZ cohort, POLE EDMs (2.6%) were significantly associated with younger age (*p =* 0.018) and localized in the left colon (*p =* 0.001). POLE non-EDMs were significantly associated with MSI-high status (*p <* 0.001) and localization in the right colon (*p =* 0.001). In the TCGA cohort, the tumor mutation burden (TMB) of both POLE EDM tumors (*p <* 0.001) and POLE non-EDM tumors (*p <* 0.001) was significantly higher than that of POLE wild-type (WT) tumors. A similar trend was observed in the ZZ cohort, although there were no significant differences. In the ZZ cohort, the POLE EDM group had higher progression-free survival (PFS) (*p =* 0.002) and overall survival (OS) (*p =* 0.042) than the POLE non-EDM group and POLE WT group. We also report one CRC patient harboring a germline POLE mutation who received camrelizumab and exhibited long-term stable disease.

**Conclusion:** Both POLE-EDMs and POLE non-EDMs were associated with significantly increased TMB in CRC and may be biomarkers for CRC treatment and prognosis. Current evidence does not support an effect of POLE non-EDMs on PFS and OS. A significant association between POLE EDMs and improved PFS and OS may exist, but future studies with larger sample sizes are needed. Entire coding region of the POLE gene should be screened.

## Introduction

The global incidence and mortality of colorectal cancer (CRC) rank first among gastrointestinal cancers (Sung et al., 2021). The poor prognosis of CRC is mainly due to its insidious onset, as approximately 25% of patients have metastasized CRC at the time of diagnosis, resulting in limited treatment options (Andre et al., 2015; Bryan et al., 2018). CRC is a highly heterogeneous cancer that develops mainly by affecting the expression and behavior of genes related to cell growth and differentiation (Fearon, 2011). In recent years, increasing studies have indicated that mutations in the DNA polymerase gene POLE mutation may be important for guiding CRC management, and are a potential biomarker for treatment and prognosis (Huhns et al., 2020).

The nuclear DNA replication-repair-associated polymerases Pol α, Polδ and Polε all belong to the polymerase B family (Doublie and Zahn, 2014). During replication, the main function of POLε is to lengthen the leading strand. The catalytic subunit of POLε has 5′ to 3′ DNA polymerase activity and 3′ to 5′ exonuclease activity and is capable of the timely removal of erroneous bases generated during replication to ensure the fidelity of DNA replication. This catalytic subunit is encoded by POLE (Henninger and Pursell, 2014). In 2012, The Cancer Genome Atlas (TCGA) exome sequencing project conducted a complete genome analysis of 224 CRC cases and showed that POLE mutation is closely related to an ultra-hypermutated phenotype (TMB >100 mut/Mb) (Cancer Genome Atlas, 2012). Subsequently, several studies have shown that CRC patients carrying POLE mutations often have TMB and infiltration of immune cells in tumors (Forgó et al., 2020; Picard et al., 2020). The aggregation of epitopes in tumors makes them more susceptible to immune checkpoint inhibitors (ICIs). To date, microsatellite instability-high (MSI-H)/deficient mismatch repair (dMMR) is the only widely recognized specific biomarker related to the positive effect of ICIs in CRC treatment (Andre et al., 2020). However, only 5% of CRC patients have MSI-H/dMMR (Battaglin et al., 2018). POLE mutations have the potential to serve as a specific biomarker to screen for candidates who may benefit from ICIs. In addition, similar to MSI in nonmetastatic CRC, POLE mutations also imply lower recurrence and metastasis rates. For stage II CRC patients whose need for adjuvant therapy is still controversial, POLE mutations indicate a better prognosis and may be important evidence for guiding treatment decisions.

In the predictions of treatment and prognosis of CRC, somatic POLE mutations have been reported to be a promising candidate biomarker. However, most studies have focused on POLE exonuclease domain mutations (EDMs) or individual mutation points. The significance of POLE non-EDMs in CRC remains unclear. Thus, this retrospective study investigated the clinical characteristics and prognostic value of POLE mutation subtypes in a real-world dataset. A similar analysis was carried out in a TCGA dataset, and the results of the two cohorts are compared and discussed.

## Methods

### Patients

The Chinese cohort included 272 CRC patients treated at The First Affiliated Hospital of Zhengzhou University (ZZ cohort) between January 2016 and December 2020. The latest follow-up date was 1 March 2021. All patients were pathologically diagnosed with primary CRC by tissue biopsy and underwent NGS. Ethics committee approval was obtained from the institutional research ethics board (NO. 2021-KY-1040-002). Data from 499 CRC patients in the TCGA database (PanCancer Atlas) (TCGA cohort) were downloaded (15 January 2022) and included in the statistical analysis (http://www.cbioportal.org/). Patients with insufficient information, including POLE status and follow-up information, were excluded. The following factors were extracted for statistical analysis: age, sex, MSI status, pathology, tumor location, tumor differentiation, clinical stage at the time of diagnosis, depth of tumor invasion, lymph node metastases, and hazard factors.

### DNA sequencing

The genomic profiling was conducted by a hybridization capture-based NGS assay using a commercial panel consisting of 520 cancer-associated genes (OncoScreen Plus, Burning Rock Biotech), spanning 1.64 Mb of the human genome (Wang et al., 2022). Tissue DNA was fragmented using Covaris M220 (Covaris, MA, United States) followed by end repair, adapter ligation and purification of fragments with sizes between 200 and 400 base pairs. Fragment size and quality were assessed with high-sensitivity DNA kit using Bioanalyzer 2100 (Agilent Technologies, CA, United States). Subsequently, the Indexed samples were sequenced on the NovaSeq 6000 platform (Illumina, Inc., CA, United States) with 150-base pair read lengths.

Sequence data were analyzed using the Burning Rock analysis system. Concisely, raw reads were aligned to the reference human genome (hg19) using Burrows-Wheeler Aligner (version 0.7.10). Variant calling was implemented using VarScan (version 2.4.3) with the following filtering steps to retain high-confidence variants: loci with depths ≥100, at least eight supporting reads for single nucleotide variations (SNVs), at least two and five supporting reads for Indel variants. Single nucleotide polymorphisms (SNPs) were all removed.

### TMB and MSI calculation

TMB was defined as the number of non-synonymous variants per megabase of genome examined, and was estimated using the OncoScreen Plus panel (OncoScreen plus, Burning Rock, Guangzhou, China) with a total size of 1.003 Mb of coding regions. Hotspot variants, copy number variations, structural variants, and germline SNPs are not counted.

MSI status of tumor and plasma samples was determined using a read-count-distribution-based approach that utilizes a given set of repeat lengths of coverage as the prime characteristic of each microsatellite locus. A locus is classified as unstable if more than 30% of the total number of microsatellite markers in the sample is below this threshold.

### Statistical analysis

Overall survival (OS) was defined as the time from histological diagnosis of CRC to death. Progression-free survival (PFS) was defined as the time from first-line therapy to the first tumor progression or recurrence. The end date was defined as the date of the last follow-up visit if there was no cancer recurrence or death. Continuous variables are described as the mean and standard deviation or the median and the interquartile range. Categorical variables are described with frequencies and percentages. The continuous variables (age at diagnosis, TMB) were compared among POLE EDM, POLE non-EDM and POLE WT groups with Kruskal-Wallis tests. Pairwise comparisons between three groups were performed using the Bonferroni correction method, and an adjusted *p* value < 0.05 was considered statistically significant. The same method was used to compare TMB levels among POLE non-EDM (MSI-L/MSS), POLE WT (MSI-H) and POLE WT (MSI-L/MSS) groups. The categorical variables were compared among POLE EDM, POLE non-EDM and POLE WT groups with Chi-square test and Fisher’s exact test. Survival function curves were generated using the Kaplan-Meier method (Ying et al., 2021). Survival differences among groups were evaluated by the log-rank test. Univariate and multivariate Cox regression models were employed to evaluate the prognostic value of POLE mutations for OS and PFS(Burke et al., 2017). All statistical analyses were performed with SPSS version 23.0 software (IBM, Chicago, IL). A two-tailed *p* value < 0.05 was considered statistically significant.

## Results

### Molecular characteristics of POLE mutations

**FIGURE 1:**
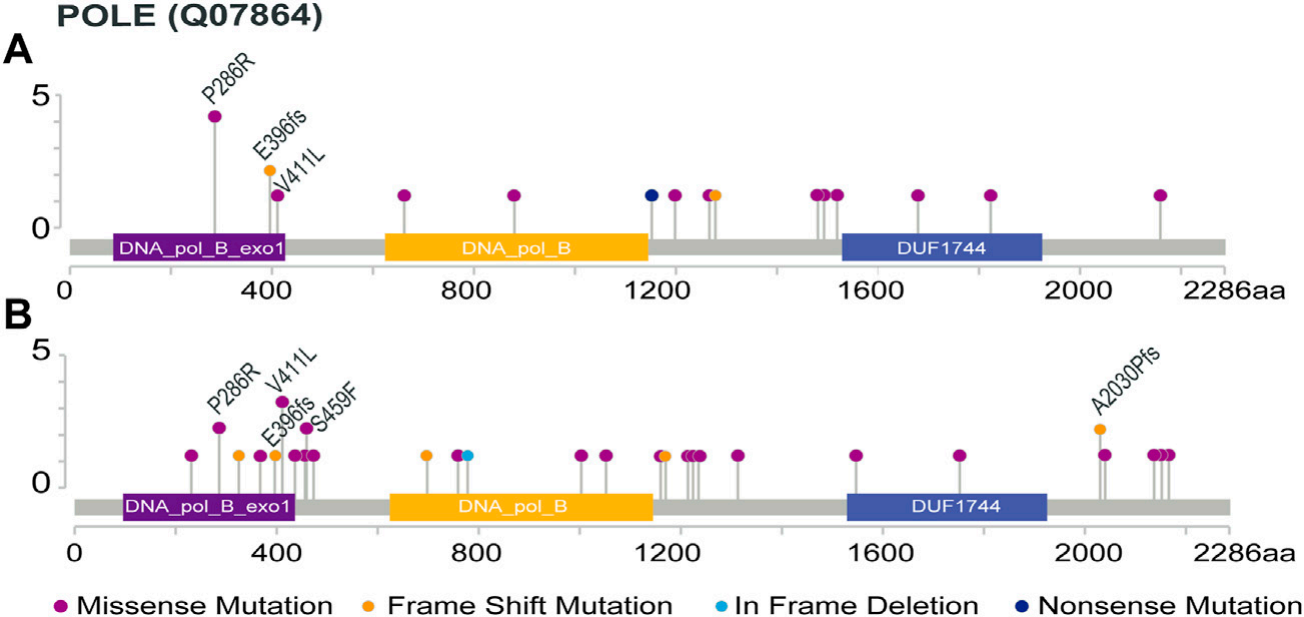
Protein distribution of POLE mutations. POLE ED including 86 to 427 amino acids (http://pfam.xfam.org/protein/DPOE1_HUMAN). **(A)** POLE mutations in the ZZ cohort, except 2 cases with intronic deletion in exon 25 and 1 case with long fragment insertion in exon 29 (the specific amino acid sites are unknown); **(B)** POLE mutations in the TCGA cohort. Recurring protein changes are labeled.

The protein distribution of POLE mutations is shown in Figure 1. In the ZZ cohort, the somatic POLE mutation rate was 7.7% (21 out of 272), including 2.6% (7 out of 272) of POLE EDMs and 5.1% (14 out of 272) of POLE non-EDMs. Five of the seven POLE EDMs were known pathogenic mutations (V411 L in 1 case, P286R in 4 cases). A mutation of unknown significance (E396 fs) was detected in 2 cases (Figure 1A). The location and genetic characteristics of each POLE mutation are shown in Table 1. Compared with POLE WT tumors, POLE non-EDM tumors were mainly MSI-H (*p* < 0.001). Most POLE EDM tumors were MSI-L/MSS; however, the difference was not significant.

**TABLE 1:** POLE variants in the ZZ cohort.

Patient ID	Exon	Protein change	Nucleotide substitution	Mutation type	MSI statue	EDM
1	23	T880M	c.2639C>T	Missense mutation	MSI-H	N
2	38	R1679C	c.5035C>T	Missense mutation	NA	N
3	13	**V411L**	c.1231G>C	Missense mutation	MSS	Y
4	9	**P286R**	c.857C>G	Missense mutation	MSS	Y
5	31	E1266K	c.3796G>A	Missense mutation	MSS	N
6	36	R1519H	c.4556G>A	Missense mutation	MSI-H	N
7	12	**E396fs**	c. (1186_1185)	Frameshift mutation	MSI-H	Y
8	9	**P286R**	c.857C>G	Missense mutation	MSS	Y
9	46	R2159C	c.6475C>T	Missense mutation	MSS	N
10	25	—	c.2865-7_2865-4del (AAAA)	Intronic deletion	MSI-H	N
11	55	A1493T	c.4477G>A	Missense mutation	MSI-H	N
12	30	A1198S	c.3592G>T	Missense mutation	MSI-H	N
13	18	N662D	c.1984A>G	Missense mutation	MSI-H	N
14	25	—	c.2865-7_2865-4del (AAAA)	Intronic deletion	MSI-H	N
15	12	**E396fs**	c. (1186_1185)	Frameshift mutation	MSI-H	Y
16	31	K1278del	c.3832_3834delAAG	In-frame deletion	MSS	N
17	9	**P286R**	c.857C>G	Missense mutation	MSS	Y
18	9	**P286R**	c.857C>G	Missense mutation	MSS	Y
19	40	R1823S	c.5467C>A	Missense mutation	MSI-H	N
20	34	P1481R	c.4442C>G	Missense mutation	MSS	N
21	29	—	—	Long fragment insertion	MSI-H	N

POLE EDMs are in bold.

In the TCGA cohort, the somatic POLE mutation rate was 6.6% (33 out of 499), including 1.8% (9 out of 499) of POLE EDMs and 4.8% (24 out of 499) of POLE non-EDMs. The 9 POLE EDMs comprised 5 known pathogenic POLE mutations (P286R, 2 cases; V411 L, 3 case). E396fs was also detected in one case (Figure 1B). Compared with POLE WT tumors, POLE non-EDM tumors were mainly MSI-H, and POLE EDM tumors were mainly MSI-L/MSS (*p* < 0.001).

In the TCGA cohort, compared with POLE WT tumors, both POLE EDM tumors (median TMB = 115.3 mut/Mb, *p <* 0.001) and POLE non-EDM tumors (median TMB = 64.2 mut/Mb, *p <* 0.001) exhibited a significantly increased TMB. A similar trend was observed in the ZZ cohort; however, in the pairwise comparisons, the Bonferroni corrected *p* values indicated no significant difference between each pair of groups (*p* > 0.05).

Given that POLE non-EDM tumors are mostly MSI-H (ZZ cohort *p* < 0.001; TCGA cohort *p* < 0.001), this study further compared the TMB level among the POLE non-EDM (MSI-L/MSS), POLE WT (MSI-H) and POLE WT (MSI-L/MSS) subgroups to explore whether the high TMB in the POLE non-EDM group should be attributed to POLE non-EDM or MSI-H status. Since only 1 case with POLE non-EDM (MSI-L/MSS), in the ZZ cohort, the difference of TMB levels among groups including POLE non-EDM (MSI-L/MSS), POLE WT (MSI-H) and POLE WT (MSI-L/MSS) were only explored in the TCGA cohort. The results showed that both the POLE non-EDM (MSI-L/MSS) group (median TMB = 93.2 mut/Mb, *p* = 0.015) and the POLE WT (MSI-H) group (median TMB = 36.4 mut/Mb) (*p* < 0.001) had significant higher TMB levels than the POLE WT (MSI-L/MSS) group (median TMB = 3.3 mut/Mb). The first two groups had similar TMB levels (*p* = 0.613), and both tended to be hypermutated phenotypes (Table 2).

**TABLE 2:** Clinicopathological characteristics according to POLE mutation status.

Characteristics	ZZ cohort				TCGA cohort			
	POLE EDM (*n* = 7)	POLE non-EDM (*n* = 14)	POLE WT (*n* = 251)	*P*	POLE EDM (*n* = 9)	POLE non-EDM (*n* = 24)	POLE WT (*n* = 466)	*P*
Age, median (range)	40.0 (34.0–52.0)	49.0 (35.75–59.5)	53.0 (46.0–64.0)	0.018	65.0 (51.5–76.5)	59.0 (49.0–70.8)	68.0 (57.0–75.0)	0.183
TMB (Mut/mb), median (range)	175.9 (28.0--)	24.0 (19.6–40.2)	8.6 (5.4–14.6)	0.012	115.3 (68.4–188.5)	64.2 (39.3–98.3)	3.4 (2.7–4.9	<0.001
Sex				0.455				0.013
Male	4 (57.1%)	10 (71.4%)	134 (53.4%)		8 (88.8%)	8 (33.3%)	248 (53.2%)	
Female	3 (42.9%)	4 (28.5%)	117 (46.6%)		1 (11.1%)	16 (66.6%)	218 (46.7%)	
MSI status				<0.001				<0.001
MSI-H	2 (28.6%)	9 (64.2%)	29 (11.6%)		4 (44.4%)	16 (66.6%)	47 (10.0%)	
MSI-L/MSS	5 (71.4%)	4 (28.5%)	212 (84.5%)		5 (55.5%)	7 (29.1%)	417 (89.4%)	
Unknown	0	1 (7.1%)	10 (4.0%)		0	1 (4.1%)	2 (0.4%)	
Pathology				0.050				0.004
Adenocarcinoma	5 (71.4%)	13 (92.8%)	234 (93.2%)		8 (88.8%)	16 (66.6%)	421 (90.3%)	
Mucinous adenocarcinoma	2 (28.5%)	1 (7.1%)	13 (5.1%)		1 (11.1%)	8 (33.3%)	45 (9.6%)	
Others	0	0	4 (1.5%)		0	0	0	
Location				0.001				0.112
Left colon	6 (85.7%)	5 (35.7%)	68 (27.0%)		1 (11.1%)	2 (8.3%)	105 (22.5%)	
Right colon	1 (14.2%)	7 (50.0%)	64 (25.4%)		4 (44.4%)	12 (50.0%)	145 (31.1%)	
Rectum	0	2 (14.2%)	119 (47.4%)		2 (22.2%)	3 (12.5)	125 (26.8%)	
Unknown	0	0	0		2 (22.2%)	7 (29.2%)	91 (19.5%)	
Grade				0.062				—
G2	3 (42.9%)	9 (64.2%)	198 (78.9%)		—	—	—	
G3	3 (42.9%)	2 (14.2%)	34 (13.5%)		—	—	—	
Unknown	1 (14.3%)	3 (21.4%)	19 (7.6%)		—	—	—	
Stage				0.536				0.207
I	1 (14.3%)	1 (7.1%)	20 (8.0%)		0	4 (16.6%)	86 (18.4%)	
II	3 (42.9%)	3 (21.4%)	51 (20.3%)		6 (66.6%)	13 (54.1%)	165 (35.4%)	
III	2 (28.6%)	3 (21.4%)	56 (22.3%)		2 (22.2%)	4 (16.6%)	141 (30.2%)	
IV	1 (14.3%)	7 (50.0%)	118 (47.0%)		0	2 (8.3%)	64 (13.7%)	
NA	0	0	6 (2.4%)		1 (11.1%)	1 (4.1%)	0	
Depth of tumor invasion				0.744				0.334
T1	0	0	2 (0.8%)		0	1 (4.1%)	16 (3.4%)	
T2	1 (14.3%)	1 (7.1%)	52 (20.7%)		1 (11.1%)	4 (16.6%)	87 (18.6%)	
T3	5 (71.4%)	8 (57.1%)	143 (57.0%)		7 (77.7%)	13 (54.1%)	318 (68.2%)	
T4	1 (14.3%)	2 (14.2%)	25 (10.0%)		1 (11.1%)	6 (25.0%)	45 (9.6%)	
NA	0	3 (21.4%)	29 (11.6%)		0	0		
Lymph node metastases				0.728				
N0	5 (71.4%)	6 (42.8%)	94 (37.5%)		7 (77.7%)	18 (75.0%)	264 (56.6%)	0.305
N1	1 (14.3%)	3 (21.4%)	54 (21.5%)		2 (22.2%)	3 (12.5%)	120 (25.7%)	
N2	1 (14.3%)	3 (21.4%)	72 (28.7%)		0	3 (12.5%)	81 (17.3%)	
Unknown	0	2 (14.2%)	31 (12.4%)		0	0	1 (0.2%)	
Hazard factor				0.181				—
no	6 (85.7%)	7 (50.0%)	84 (33.5%)		—	—	—	
Perineural invasion	0	0	37 (14.7%)		—	—	—	
Vascular carcinoma embolus	1 (14.3%)	1 (7.1%)	44 (17.5%)		—	—	—	
Both	0	2 (14.2%)	43 (17.1%)		—	—	—	
Unknown	0	4 (28.5%)	43 (17.1%)		—	—	—	

### Clinicopathological features of CRC with somatic POLE mutations

In the ZZ cohort, the POLE EDM group had younger age at diagnosis (*p =* 0.018) and more frequent left-sided tumor localization (*p =* 0.002). Right-sided tumor localization was more frequent in the POLE non-EDM group (*p =* 0.001). Most POLE EDM tumors were diagnosed at an early stage and had a low risk of recurrence. Among POLE EDM tumors, 3 at stage II (42.9%, *p* = 0.536), 5 at pT3 (71.4%, *p* = 0.744), 5 at N0 (71.4%, *p* = 0.728), 6 at M0 (85.7%, *p* = 0.247) and 6 had no hazard factors (85.7%, *p* = 0.181).

In the TCGA cohort, POLE EDM mostly occurred in male patients (*p =* 0.013). Patients with POLE non-EDM tumors more frequent had right-sided tumor localizations (*p* = 0.010) and adenocarcinoma histology (*p* = 0.004). Among POLE EDM tumors, there were 6 at stage II (66.6%, *p* = 0.207), 7 at pT3 (77.7%, *p* = 0.334),7 at N0 (77.7%, *p* = 0.305) and 8 at M0 (88.9%, *p* = 0.805). The detailed clinicopathological features of patients in the ZZ cohort and TCGA cohort are summarized in Table 2.

### Prognostic value of POLE mutations

**FIGURE 2:**
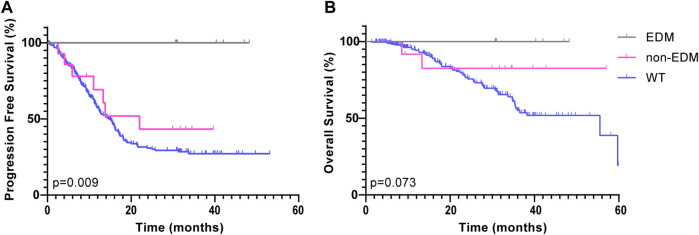
Kaplan-Meier survival curves of patients in the ZZ cohort according to POLE mutation status. PFS **(A)** and OS **(B)** of POLE EDMs, non-EDMs and WT patients in the ZZ cohort.

All patients were divided into 3 subgroups: the POLE EDMs, POLE non-EDMs and POLE WT groups. In the ZZ cohort, the 272 CRC patients were followed for a median of 16.8 months. Since no patients in the POLE EDM group had progressed by the last follow-up, the median PFS was not reached. Based on the stratified log-rank test, the PFS rate of the POLE EDM group was significantly higher than that of the POLE non-EDM (median = 22.0 months, χ^2^ = 5.407, *p =* 0.020) and POLE WT groups (median = 14.6 months, χ^2^ = 8.830, *p =* 0.003) (Figure 2A). The OS of the POLE EDM group and POLE non-EDM group were not reached. There was no significant difference in OS among these three groups (*p =* 0.056) (Figure 2B).

**FIGURE 3:**
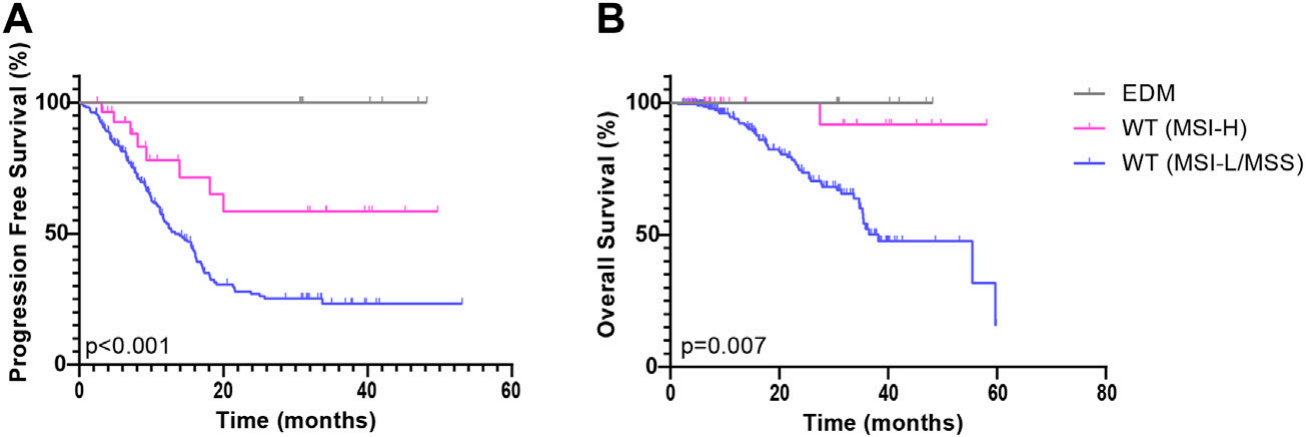
Kaplan-Meier survival curves of patients in the ZZ cohort according to POLE mutation and MMR status. PFS **(A)** and OS **(B)** of patients in the POLE EDM, POLE WT (MSI-H) and POLE WT (MSI-L/MSS) groups.

POLE WT group was further subdivided into the POLE WT (MSI-L/MSS) subgroup and the POLE WT (MSI-H) subgroup. Based on the Kaplan-Meier analysis, both the POLE EDM group (χ^2^ = 9.845, *p =* 0.002) and the POLE WT (MSI-H) group (χ^2^ = 7.036, *p =* 0.008) showed improved PFS compared to the POLE WT (MSI-L/MSS) group (median = 13.3 months) (Figure 3A). In analyses that used OS as the end point, the POLE EDM group (χ^2^ = 4.125, *p =* 0.042) and the POLE WT (MSI-H) group (χ^2^ = 6.032, *p =* 0.014) also showed better outcomes than the POLE WT (MSI-L/MSS) group (median = 38.2 months) (Figure 3B). The prognosis of the POLE EDM group and POLE WT (MSI-H) group was similar. The PFS and OS of the POLE EDM group and POLE WT (MSI-H) group were not reached.

**FIGURE 4:**
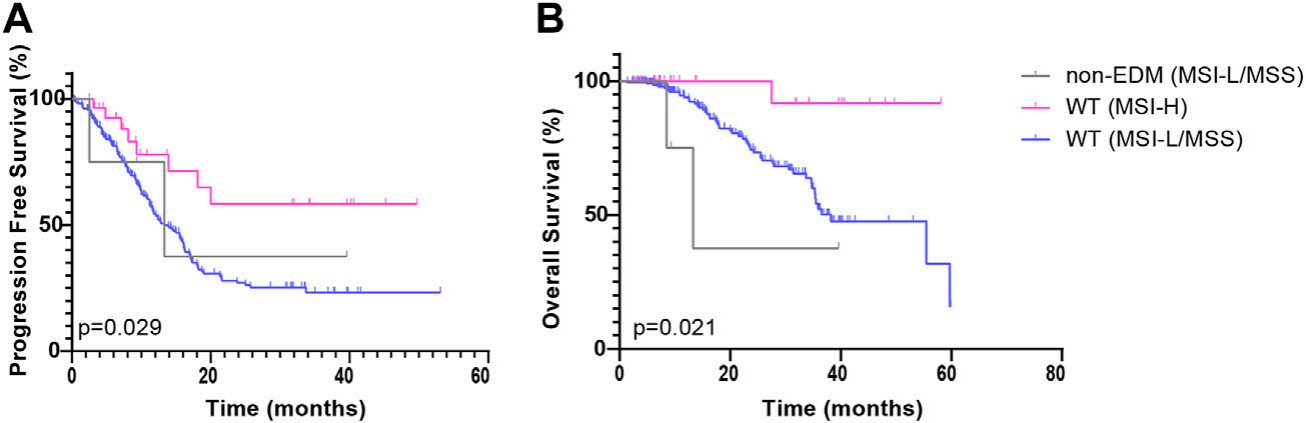
Kaplan-Meier survival curves of patients in the ZZ cohort according to POLE mutation and MMR status. PFS **(A)** and OS **(B)** of patients in the POLE non-EDM, POLE WT (MSI-H) and POLE WT (MSI-L/MSS) groups.

In this study, POLE non-EDM tumors were mostly MSI-H (ZZ cohort *p* < 0.001; TCGA cohort *p* < 0.001). To exclude the effect of the interaction of MSI and POLE non-EDMs on PFS, the Kaplan—Meier survival curves of the POLE non-EDM (MSI-L/MSS) group, POLE WT (MSI-H) group, and POLE WT (MSI-L/MSS) group were compared with pairwise comparisons to explore the influence of POLE non-EDMs alone. In the ZZ cohort, PFS exhibited similar trends in the POLE non-EDM (MSI-L/MSS) group (median = 13.3 months, χ^2^ = 0.131, *p* = 0.718) and POLE WT (MSI-L/MSS) group (median = 13.3 months) (Figure 4A). Similarly, here was no significant difference between the POLE non-EDM (MSI-L/MSS) group (median = 13.3 months, χ^2^ = 1.361, *p =* 0.243) and the POLE WT (MSI-L/MSS) group (median = 38.2 months) on OS (Figure 4B). With the univariate and multivariate Cox regression models, POLE EDM and POLE non-EDM were both prognostic protective factors (HR<1) without statistical significance levels (Table 3, Supplementary Table S1). Distant metastasis and advanced clinical stage (stage III-IV) were independent risk factors for shortened PFS while age ≥60 and poor differentiation (G3) were independent risk factors for shortened OS.

**TABLE 3:** Multivariate Cox regression models of PFS and OS.

	ZZ cohort			TCGA cohort		
	HR	95%CI	*P*	HR	95%CI	*P*
PFS						
POLE						
WT	1		0.617	1		0.445
EDM	0	0-3.638E+223	0.962	2.105	0.662–6.687	0.207
Non-EDM	0.477	0.109–2.089	0.326	1.102	0.475–2.558	0.821
OS						
POLE						
WT	1		1	1		0.090
EDM	0	0.000-#	0.984	2.991	0.930–9.617	0.066
Non-EDM	0	0.000-#	0.984	1.766	0.755–4.131	0.189

(0000- #): The mortality was extremely low in the POLE EDM group and POLE non-EDM, group of the ZZ cohort, so the upper 95% CI, could not be computed.

In the TCGA cohort, the median follow-up periods of 499 CRC patients was 22.0 months. The similar analyses were also performed among these three subgroups in the TCGA cohort; however, there were no significant differences on PFS and OS among the groups. With the univariate and multivariate Cox regression models of the TCGA cohort, POLE EDM and POLE non-EDM were both prognostic risk factors (HR>1) without statistical significance levels (Table 3, Supplementary Table S2). Lymph node metastasis and pT4 were independent risk factors for shortened PFS. Distant metastasis, pT4 and age >60 years are independent risk factors for shortened OS.

### A patient with an inherited germline POLE mutation treated with camrelizumab

Polymerase proof-reading associated polyposis and Lynch-like syndrome are inherited cancer susceptibility syndromes associated with germline POLE mutations. (Elsayed et al., 2015; Vande Perre et al., 2019). Such patients often progressively develop CRC or extraintestinal tumors (Bellido et al., 2016). Identifying germline POLE mutations may help to understand the pathogenesis of CRC, reduce the morbidity and mortality, and guide treatment. The germline POLE mutations identified in CRC patients published from 2017 to 2020 are summarized in Table 4. There were two relatively rare cases in which p. V411L was previously described as a somatic hotspot alteration and p. V474I was located outside the ED. This study also reports a rare case.

**TABLE 4:** Summary of germline POLE mutations in colorectal cancer reported in published articles (2017–2020).

Protein change	Nucleotide substitution	MSI/MMR statue	EDM	Family history	ACMG	Ref
T457M	c.1370C>T	NA	Y	NA	Uncertain significance	Siraj et al. (2020)
S314A	c.940T>G	NA	Y	Y	Uncertain significance	
H342Y	c.1024C>T	pMMR	Y	NA	Uncertain significance	
G395E	c.1184G>A	pMMR	Y	NA	Uncertain significance	
N363K	c.1089C>A	MSS	Y	Y	Pathogenicity	Vande Perre et al. (2019)
V411L	c.1231G>C	MSS	Y	NA	likely pathogenic	Wimmer et al. (2017)
V474I	c.1420G > A	pMMR	N	NA	likely pathogenic	Esteban-Jurado et al. (2017)
E277G	c.830A>G	pMMR	Y	Y	Pathogenicity	Rosner et al. (2018)
T278K	c.833C>A	pMMR	Y	Y	Pathogenicity	Castellsague et al. (2019)
L424V	c.1270C>G	NA	Y	NA	Pathogenicity	Elsayed et al. (2019)
L283*	c.849C > T	MSS	Y	NA	likely pathogenic	Lasabova et al. (2019)
Met294Arg	c.881T>G	pMMR	Y	Y	likely pathogenic	Mur et al. (2020)
Ala426Val	c.1277C>T	pMMR	Y	Y	Uncertain significance

* : A silent mutation in codon p.L283 = (CTC >CTT).

**FIGURE 5:**
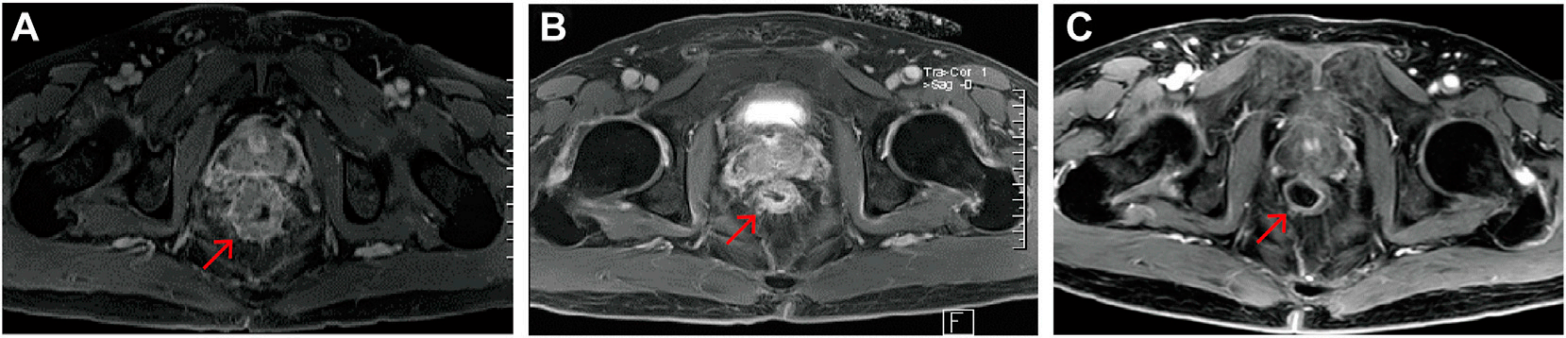
Rectal magnetic resonance images of the POLE-mutant rectal cancer patient receiving camrelizumab and anlotinib. **(A)** Pre-immunotherapy; **(B)** 7 months post—immunotherapy; **(C)** 18 months post—immunotherapy. Red arrows indicate the same rectal tumor lesion.

**FIGURE 6:**
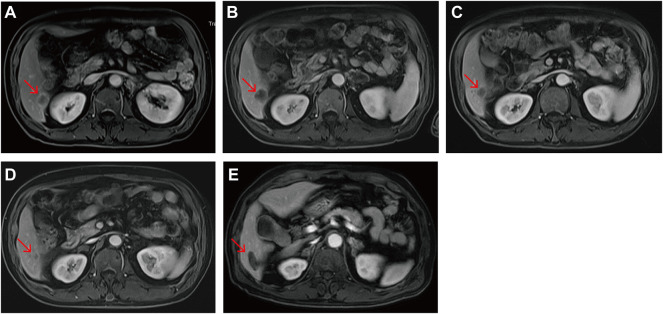
Liver metastasis magnetic resonance images of the POLE-mutant rectal cancer patient receiving camrelizumab and anlotinib. **(A)** Pre-immunotherapy; **(B)** 1 month post—immunotherapy; **(C)** 3 months post—immunotherapy; **(D)** 7 months post—immunotherapy; **(E)** 9 months post—immunotherapy. The image of the metastasis 2 months after argon-helium cryoablation. Red arrows indicate the same liver metastases.

A male patient with abdominal pain, abdominal distention, and difficulty defecating was referred to our center in December 2019. Medical imaging examination and tissue biopsy suggested bowel obstruction and rectal adenocarcinoma with multiple lymph node metastases. He received first-line treatment with an oxaliplatin plus capecitabine regimen. He developed adrenal metastasis 3 months later and was treated with bevacizumab. However, rectal occupation progressed soon after this addition, so the above regimen was stopped. Treatment with “FOLFIRI + bevacizumab” began on 16 April 2020; however, the effect was poor. The tumor continued to progress, and the patient presented with liver metastasis 2 months later. NGS results of a 41-gene panel suggested the presence of a POLE mutation (exon 45, S2084 fs), KRAS mutation (G12S), TP53 (R2084 fs) and MSS. The administration of anlotinib and camrelizumab began on 8 June 2020 and was continued until the last follow-up (6 December 2021), with no progression observed (PFS >18 months) (Figures 5, 6). The POLE mutation was an inherited germline mutation located outside the ED; this variant has not been previously identified in a large population database. According to the ACMG 2015 guidelines, this variant was evaluated as a hereditary variant with possible pathogenicity. The patient had MSS but received sustained long-term benefit from immunotherapy. It is believed that POLE mutation may be used to predict the response to ICIs.

## Discussion

This study included mutations inside and outside of the POLE exonuclease domain. We aimed to explore the molecular pathological features and prognostic value of different POLE mutation subtypes. As previously reported, in the Asian population, POLE EDMs are mainly found in the left colon and relatively young CRC patients (Hino et al., 2019; Hu et al., 2021), whereas POLE non-EDMs are more common in the right colon.

Somatic POLE mutations were evenly located throughout the POLE gene with no apparent tendency to cluster as shown in Figure 1. In the ZZ cohort, the frequency of POLE EDMs was 2.6%, while the frequency of POLE non-EDMs was 5.1%; in the TCGA cohort, the frequencies were 1.8% and 4.8%, respectively. This finding is consistent with the previously reported frequency of POLE mutations in CRC (Campbell et al., 2017). Although the frequency of POLE mutations is low, its unique high immunogenicity has attracted widespread attention.

Tumors harboring POLE EDMs often manifest with a high TMB, which is associated with an enhanced intertumoral immune response and better outcome (Llosa et al., 2015). This discovery was first reported in the TCGA whole-exome sequencing project in 2012 and is a critical first step for moving treatment of toward precision therapy. In addition, some tumors harboring only POLE non-EDMs also exhibited elevated mutation burdens, such as C810 and E978. This study showed that both POLE EDMs and POLE non-EDMs were associated with significantly increased TMB (Table 2). Since the POLE EDM tumors in this study were mostly MSI-L/MSS, and the POLE non-EDM tumors were mostly MSI-H, we analyzed the data again after excluding the interference of MSI status and still reached the same conclusion. POLE EDM tumors tended to have ultra-hypermutated phenotypes (TMB>100 mut/Mb), and POLE non-EDM tumors tended to have hypermutated phenotypes (TMB>10 mut/Mb). Although consistent with previous reports that POLE EDMs are predominantly MSS, this study identified 3 cases of CRC harboring both POLE E396fs and MSI-H (ZZ cohort, 2 cases; TCGA cohort, 1 case) (Stenzinger et al., 2014; Kawai et al., 2021). All 3 cases were stage II CRC with prolonged PFS. The significance of this mutation merits further study. This study indicated that mutation location is not a determining factor for the predictive value of POLE mutations. Thus, it is necessary to thoroughly assess POLE mutations throughout the coding region.

In tumors with MSI/dMMR or POLE mutations, the production of new antigens is caused by a large accumulation of nonsynonymous substitution and/or frameshift mutations. Major histocompatibility complexs can present these new antigens to the immune system, thereby enhancing the immune system’s attack on tumor cells. In recent years, several patients with both POLE EDMs and MSS have been reported to obtain clinical benefit from ICI treatment (Guerra et al., 2017; Keenan et al., 2021). A study of a cohort of 295 patients with stage II CRC indicated that POLE mutant tumors have significantly elevated mutation levels (Domingo et al., 2016). These patients have a better prognosis and may not require adjuvant treatment. Studies have indicated that the predicted amount of new antigens in MSI/dMMR tumors is 10–50 times those in MSS tumors, and in POLE mutant tumors produce 15 times the amount of new antigens compared to that of MSI/dMMR tumors (Shinbrot et al., 2014; Howitt et al., 2015). Therefore, the prognosis and treatment response of CRC patients with POLE mutations may be improved and enhanced.

MSI and POLE mutations have similar effects on tumors. To exclude the influence of MSI status and thus determine the prognostic value of POLE mutation itself, this study conducted 3 subgroup analyses according to POLE mutation and MSI status. Additionally, the prognostic value of MSI status and POLE mutation was compared.

First, this study divided all patients into three groups: the POLE EDM, POLE non-EDM and POLE WT groups. In the ZZ cohort, we found that POLE EDM tumors were less prone to recurrence or progression than POLE WT tumors (Figure 2A). POLE non-EDM tumors did not show a PFS advantage. Moreover, no difference in OS was observed among the groups (Figure 2B). Subsequently, we divided the patients into POLE EDM, POLE WT (MSI-H) and POLE WT (MSI-L/MSS) subgroups. In the ZZ cohort, POLE EDM and POLE WT (MSI-H) tumors had better OS and PFS outcomes than POLE WT (MSI-L/MSS) tumors (Figure 3). POLE EDMs and MSI-H status had similar roles in improving the prognosis of CRC. Finally, we divided the patients into POLE non-EDM (MSI-L/MSS), POLE WT (MSI-H) and POLE WT (MSI-L/MSS) subgroups. POLE non-EDM tumors did not show improvement or deterioration of PFS or OS in the ZZ cohort (Figure 4). Based on the above subgroup analyses, POLE EDMs and MSI-H statue improve clinical outcomes to a similar degree. Currently, POLE non-EDMs do not demonstrate this beneficial effect.

In the univariate and multivariate Cox regression models, POLE EDMs and POLE non-EDMs were both protective factors for PFS and OS prolongation (HR<1) in the ZZ cohort but did not reach statistical significance levels (Table 3). We considered that the accuracy and validity of the Cox regression model was reduced due to the high proportion of censored data for most patients who did not reach the clinical outcome of PFS or OS.

In this study, the above 3 subgroup analyses were also performed in the TCGA cohort; however, POLE mutations did not show an effect on the PFS or OS outcomes. Paradoxically, POLE mutation may be a risk factor for reduce PFS and OS in the TCGA cohort (HR > 1). It should be noted that the cases in the TCGA cohort were diagnosed from 1998 to 2013. However, the clinical application of ICIs has only gradually been realized in the past 5 years. POLE mutations and MSI-H statue are both factors closely related to the effect of immunotherapy. Therefore, the above contradictory results are likely related to the application of ICIs. In addition, it is worth noting that only 12 (2.4%) cases in the TCGA dataset were Asian, and the differences between ethnic groups cannot be ignored.

Somatic POLE mutations have the potential to guide personalized treatment, thereby improving clinical outcomes. The discovery of germline POLE mutations is highly important for reducing the incidence of CRC. Esteban et al. reported a germline POLE mutation (V474I) located outside the ED (Esteban-Jurado et al., 2017). This study also identified a potentially pathogenic germline POLE non-EDM (S2084 fs). This metastatic rectal cancer patient progressed rapidly after early treatment but obtained continued benefits after receiving camrelizumab and anlotinib (PFS >18 months). Interestingly, after two cycles of application of this regimen, MRI scans showed that the metastasis in the right lower lobe of the liver first increased and then gradually decreased and remained stable after continuous administration (Figure 6). We suggested the efficiency of ICI treatment should not be evaluated too soon after application due to the temporary increase in reactivity.

This study excluded the effect of MSI status on tumors and extended the scope of the study to the entire region of the POLE gene. We fully analyzed the clinico-molecular pathological features of POLE EDM tumors and POLE non-EDM tumors and the prognostic impact of POLE mutation subtypes from different aspects. In addition, this study compared the difference between the effects of POLE mutation and MSI status on CRC. Our study also had a few limitations. First, patients with POLE mutations had a high survival rate and PFS rate, and the insufficient follow-up time resulted in insufficient statistical power for some subgroups. We need to continue to closely follow-up with these patients. Second, the total number of POLE mutation was small, and additional studies are required to verify the applicability of the findings in this study. Third, racial differences in the clinical characteristics and prognosis of CRC patients with POLE mutations should be explored further in future studies.

In conclusion, both POLE EDMs and POLE non-EDMs were associated with significantly increased TMB in CRC, which is an important biomarker for CRC treatment and prognosis. It is also necessary to study the entire region of the POLE gene. POLE EDMs may be significantly associated with prolonged PFS and OS; however, the evidence is currently insufficient. Future studies need larger sample sizes to provide more data. The current data do not support the impact of POLE non-EDMs on CRC prognosis. Future studies need to eliminate the interference caused by ethnicity and treatment to analyze the specific role of POLE genes more accurately.

## Data Availability

The original contributions presented in the study are included in the article/Supplementary Material, further inquiries can be directed to the corresponding authors.

## Abbreviations

ACMG, American College of Medical Genetics and Genomics; CRC, colorectal cancer; dMMR, deficient mismatch repair; ED, exonuclease domain; EDMs, exonuclease domain mutations; ICIs, immune checkpoint inhibitors; MSI, microsatellite instability; MSS, microsatellite stability; mCRC, metastasis colorectal cancer; NA, not assessable; OS, overall survival; PFS, progression-free survival; pMMR, proficient mismatch repair; TCGA, The Cancer Genome Atlas; TMB, tumor mutation burden; 5-FU, 5-Fluorouracil; WT, wild-type; Y, yes; N, no.
