# Supplementary material for: Distinct clinical pattern of colorectal cancer patients with POLE mutations: A retrospective study on real-world data
Source: Front Genet. 2022 Nov 21;13:963964. doi: 10.3389/fgene.2022.963964 (PMC9719917; doi:10.3389/fgene.2022.963964)
Supplement: Supplementary file 1 [file DataSheet1.doc]

**Supplemental table S1 Univariate Cox regression models of PFS and OS in the ZZ cohort**

| **Characteristics** | **PFS** | | |  | **OS** | | |
| --- | --- | --- | --- | --- | --- | --- | --- |
|  | **HR** | **95%CI** | ***P*** |  | **HR** | **95%CI** | ***P*** |
| **POLE** |  |  |  |  |  |  |  |
| WT | 1 |  | 0.651 |  | 1 |  | 0.469 |
| EDM | 0 | 0-2.612E+200 | 0.957 |  | 0 | 0-. | 0.972 |
| Non-EDM | 0.698 | 0.326-1.495 | 0.355 |  | 0.412 | 0.100-1.693 | 0.219 |
| **Age (year)** |  |  |  |  |  |  |  |
| <60 | 1 |  |  |  | 1 |  |  |
| ≥60 | 0.817 | 0.573-1.165 | 0.265 |  | 1.778 | 1.065-2.967 | 0.028 |
| **Sex** |  |  |  |  |  |  |  |
| Male | 1 |  |  |  | 1 |  |  |
| Female | 1.257 | 0.899-1.759 | 0.181 |  | 1.846 | 1.099-3.101 | 0.020 |
| **Location** |  |  |  |  |  |  |  |
| Left colon | 1 |  | 0.337 |  | 1 |  | 0.526 |
| Right colon | 0.925 | 0.582-1.470 | 0.742 |  | 1.210 | 0.632-2.317 | 0.565 |
| Rectum | 1.233 | 0.826-1.839 | 0.305 |  | 0.848 | 0.454-1.584 | 0.605 |
| **TMB (mut/Mb)** |  |  |  |  |  |  |  |
| <10 | 1 |  |  |  | 1 |  |  |
| ≥10 | 0.623 | 0.363-1.069 | 0.086 |  | 0.639 | 0.201-2.030 | 0.448 |
| **MSI status** |  |  |  |  |  |  |  |
| MSI-L/MSS | 1 |  |  |  | 1 |  |  |
| MSI-H | 0.411 | 0.226-0.745 | 0.003 |  | 0.077 | 0.011-0.561 | 0.011 |
| **Pathology** |  |  |  |  |  |  |  |
| Adenocarcinoma | 1 |  |  |  | 1 |  |  |
| Mucinous adenocarcinoma | 0.741 | 0.346-1.585 | 0.439 |  | 1.303 | 0.52-3.264 | 0.573 |
| **Grade** |  |  |  |  |  |  |  |
| G1-G2 | 1 |  |  |  | 1 |  |  |
| G3 | 1.282 | 0.806-2.041 | 0.294 |  | 2.235 | 1.200-4.162 | 0.011 |
| **Depth of tumor invasion** |  |  |  |  |  |  |  |
| T1-T3 | 1 |  |  |  | 1 |  |  |
| T4 | 1.116 | 0.680-1.831 | 0.665 |  | 1.053 | 0.611-1.815 | 0.853 |
| **Distant metastasis** |  |  |  |  |  |  |  |
| No | 1 |  |  |  | 1 |  |  |
| Yes | 5.618 | 3.739-8.443 | <0.001 |  | 4.204 | 2.124-8.321 | <0.001 |
| **Lymph node metastases** |  |  |  |  |  |  |  |
| No | 1 |  |  |  | 1 |  |  |
| Yes | 2.445 | 1.614-3.702 | <0.001 |  | 9.395 | 3.351-26.342 | <0.001 |
| **Hazard factor** |  |  |  |  |  |  |  |
| No | 1 |  |  |  | 1 |  |  |
| Yes | 2.073 | 1.359-3.163 | 0.001 |  | 2.185 | 1.099-4.342 | 0.026 |
| **Stage** |  |  |  |  |  |  |  |
| Early (I-II) | 1 |  |  |  | 1 |  |  |
| Late (III-IV) | 5.990 | 3.295-10.887 | <0.001 |  | 24.185 | 3.343-174.963 | 0.002 |

Since none of the patients in the POLE EDM group reached the clinical outcome of PFS, the output HR=0 and the upper limit of the 95% CI was extremely high.

**Supplemental table S2 Univariate Cox regression models of PFS and OS in the TCGA cohort**

| **Characteristics** | **PFS** | | |  | **OS** | | |
| --- | --- | --- | --- | --- | --- | --- | --- |
|  | **HR** | **95%CI** | ***P*** |  | **HR** | **95%CI** | ***P*** |
| **POLE** |  |  |  |  |  |  |  |
| WT | 1 |  | 0.861 |  | 1 |  | 0.511 |
| EDM | 1.265 | 0.402-3.981 | 0.688 |  | 1.656 | 0.524-5.240 | 0.391 |
| Non-EDM | 1.162 | 0.542-2.493 | 0.699 |  | 1.378 | 0.639-2.974 | 0.414 |
| **Age (year)** |  |  |  |  |  |  |  |
| <60 | 1 |  |  |  | 1 |  |  |
| ≥60 | 0.802 | 0.556-1.158 | 0.239 |  | 1.823 | 1.130-2.942 | 0.014 |
| **Sex** |  |  |  |  |  |  |  |
| Male | 1 |  |  |  | 1 |  |  |
| Female | 0.783 | 0.551-1.113 | 0.173 |  | 0.970 | 0.660-1.425 | 0.877 |
| **Location** |  |  |  |  |  |  |  |
| Left colon | 1 |  | 0.657 |  | 1 |  | 0.337 |
| Right colon | 1.207 | 0.803-1.814 | 0.365 |  | 1.285 | 0.832-1.987 | 0.258 |
| Rectum | 1.152 | 0.637-2.082 | 0.639 |  | 0.809 | 0.374-1.752 | 0.591 |
| **TMB (mut/Mb)** |  |  |  |  |  |  |  |
| <10 | 1 |  |  |  | 1 |  |  |
| ≥10 | 0.866 | 0.532-1.410 | 0.563 |  | 0.965 | 0.573-1.623 | 0.893 |
| **MSI status** |  |  |  |  |  |  |  |
| MSI-L/MSS | 1 |  |  |  | 1 |  |  |
| MSI-H | 0.962 | 0.577-1.602 | 0.881 |  | 0.986 | 0.562-1.733 | 0.962 |
| **Pathology** |  |  |  |  |  |  |  |
| Adenocarcinoma | 1 |  |  |  | 1 |  |  |
| Mucinous adenocarcinoma | 0.875 | 0.483-1.585 | 0.660 |  | 1.279 | 0.728-2.246 | 0.392 |
| **Depth of tumor invasion** |  |  |  |  |  |  |  |
| T1-T3 | 1 |  |  |  | 1 |  |  |
| T4 | 4.096 | 2.660-6.307 | <0.001 |  | 3.967 | 2.448-6.430 | <0.001 |
| **Distant metastasis** |  |  |  |  |  |  |  |
| No | 1 |  |  |  | 1 |  |  |
| Yes | 4.704 | 3.189-6.937 | <0.001 |  | 3.697 | 2.388-5.722 | <0.001 |
| **Lymph node metastases** |  |  |  |  |  |  |  |
| No | 1 |  |  |  | 1 |  |  |
| Yes | 2.453 | 1.724-3.491 | <0.001 |  | 2.513 | 1.696-3.723 | <0.001 |
| **Stage** |  |  |  |  |  |  |  |
| Early (I-II) | 1 |  |  |  | 1 |  |  |
| Late (III-IV) | 2.683 | 1.869-3.851 | <0.001 |  | 2.765 | 1.835-4.167 | <0.001 |
